# 异基因造血干细胞移植治疗RASGRP2基因相关遗传性血小板功能障碍1例报告并文献复习

**DOI:** 10.3760/cma.j.cn121090-20250822-00394

**Published:** 2026-04

**Authors:** 彩丽 王, 令恒 孟, 文 夏, 思琪 龙, 常莹 方, 筱灵 梁

**Affiliations:** 贵阳市妇幼保健院/贵阳市儿童医院，贵阳 550001 Guiyang Maternal and Child Health Care Hospital, Guiyang Children's Hospital, Guiyang 550001, China

## Abstract

本文对1例经异基因造血干细胞移植（allo-HSCT）治愈的RASGRP2基因相关遗传性血小板功能障碍（IPFD）患儿进行回顾性分析，并进行相关文献复习。患儿，男，4岁8个月，因“反复鼻出血3年”就诊，完善相关检查，诊断为RASGRP2基因相关IPFD。患儿存在致死性出血风险，常规治疗效果不佳，故选择HLA全相合且未携带致病基因的同胞兄长作为造血干细胞供者，采用清髓性预处理方案，移植后第13天血小板植入，移植后第18天粒细胞及红细胞植入，移植后第22天检测嵌合率为99.7％。预处理期间患儿出现鼻出血，经鼻腔填塞及输注血小板后症状控制，血小板植入后未再发生鼻出血。随访至移植后4个月，无移植物抗宿主病、严重感染等并发症。检测嵌合率提示为完全供者型。

## Abstract (en)

This study retrospectively analyzed a 4-year-and-8-month-old boy with RASGRP2-associated inherited platelet function disorders（IPFD）who was successfully treated with allogeneic hematopoietic stem cell transplantation, along with a review of the relevant literature. The patient presented with a 3-year history of recurrent epistaxis, and following comprehensive evaluation, he was diagnosed as having RASGRP2-related IPFD. Because of life-threatening bleeding risk and poor response to conventional treatment, an HLA-matched sibling without the pathogenic variant was selected as the donor, and a myeloablative conditioning regimen was administered. Platelet engraftment was achieved on Day 13 posttransplantation, and neutrophil and erythrocyte engraftment occurred on Day 18. Donor chimerism reached 99.7% on Day 22. Epistaxis during conditioning was controlled with nasal packing and platelet transfusion, and no bleeding recurred after platelet engraftment. At the 4-month follow-up, no graft-versus-host disease or severe infection was detected, and complete donor chimerism was confirmed.

遗传性血小板功能障碍（inherited platelet function disorders，IPFD）是一类基因突变导致血小板功能异常的罕见出血性疾病，以高度异质性的皮肤黏膜出血为主要表现。随着分子生物学技术的发展，越来越多的相关基因被发现。RAS鸟嘌呤核苷酸释放蛋白2（RAS guanyl-releasing protein-2, RASGRP2）是一个位于染色体11q13.1区域的基因，包含2个非翻译外显子和15个编码外显子，该基因致病性变异导致血小板功能异常性出血。治疗以局部止血、抗纤溶药物、血小板输注为主。我院收治了1例RASGRP2基因新发突变相关血小板功能缺陷严重出血儿童，经异基因造血干细胞移植（allo-HSCT）治愈，报道如下。

## 病例资料

患儿，男，4岁8个月，籍贯为贵州省毕节市，苗族。因“反复鼻出血3年”就诊，3年来无诱因反复出现鼻出血，出血量大，止血困难，每次出血需到医院行鼻腔填塞止血。患儿每年出血2～5次，多次因“失血性贫血、缺铁性贫血、鼻出血”住院，予补充铁剂及输注红细胞治疗。患儿无反复感染，无其他部位出血。父母非近亲结婚，同胞姐姐1岁5个月时因大量鼻出血死亡，2个同胞哥哥分别为9岁、7岁，父母及哥哥均无异常出血史。查体：生长发育正常，皮肤未见出血点、瘀斑、血肿，浅表淋巴结、肝脾未扪及肿大，关节无肿胀畸形。血小板计数及形态正常，凝血功能正常，血栓弹力图正常。流式细胞术检测血小板膜糖蛋白CD41（GPⅡb）78％、CD61（GPⅢa）77.8％、CD42b（GPⅠb）73.4％，略降低，活化血小板CD62p（P-选择素）58.4％。血小板聚集试验：在二磷酸腺苷和胶原蛋白诱导下严重受损，花生四烯酸诱导的聚集未受影响。全外显子组基因检测V6（200X）：RASGRP2基因纯合变异，c.74-1G>C（p.?），根据美国医学遗传学与基因组学学会指南，该变异初步判定为致病性变异。PVS1+PM2_Supporting+PM3_Supporting（hom）：PVS1变异为零效变异（剪接突变），可能导致基因功能丧失；PM2_Supporting在正常人群数据库中的频率为未知；PM3_Supporting（hom）变异为纯合稀有变异；经验证，先证者父母该位点杂合变异，同胞大哥该位点无变异，同胞二哥该位点杂合变异。家系分析显示，符合常染色体隐性遗传规律（图1）。

**图1:**
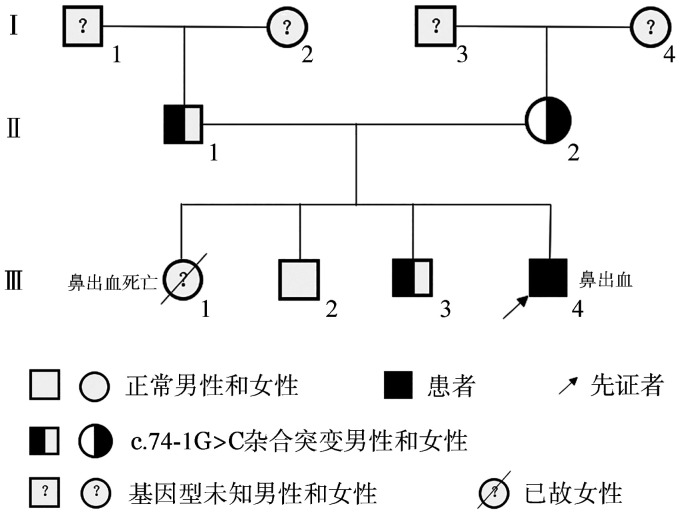
RASGRP2基因相关遗传性血小板功能障碍家系图

因患儿多次突发危及生命的大量鼻出血，多次住院常规治疗效果不佳，同胞姐姐因鼻出血死亡，家长强烈要求选择allo-HSCT。2个同胞哥哥与患儿HLA配型均12/12相合，选择未携带该基因的大哥作为造血干细胞供者。预处理方案采用白消安注射液（1 mg/kg×8次）、环磷酰胺（60 mg/kg×2次）、氟达拉滨（40 mg/m^2^×5次）、兔抗人胸腺细胞免疫球蛋白（10 mg/kg分4次），输注造血干细胞前1 d中性粒细胞绝对计数为1.75×10^9^/L，淋巴细胞计数为0.01×10^9^/L。考虑清髓程度未达到标准，加用美法仑130 mg/m^2^输注。采用环孢素、吗替麦考酚酯及短疗程甲氨蝶呤预防移植物抗宿主病（GVHD）。回输供者外周血造血干细胞计数单个核细胞计数12.6×10^8^/kg，CD34^+^细胞计数13.9×10^6^/kg。移植后第13天血小板植入，移植后第18天粒细胞、红细胞植入，移植后第22天查嵌合率为99.7％。预处理期间鼻出血1次，按压鼻腔及止血药物治疗不能止血，耳鼻喉科予明胶海绵填塞后未见活动性出血。1周后取出填塞物，仍见大量血液流出，再次填塞1周后，取出填塞物未见出血。血小板植入后未再出现鼻腔出血。

随访至移植后4个月，无鼻腔或其他部位出血，无GVHD、严重感染等并发症，监测嵌合率为完全供者型。移植后2个月、3个月复查血小板聚集试验，二磷酸腺苷诱导下聚集试验已正常，胶原蛋白诱导下仍受损。

## 讨论及文献复习

血小板是止血的关键细胞成分，正常的血小板被激活后聚集、黏附于血管损伤部位，达到止血目的。正常血小板功能依赖于关键表面受体和信号蛋白的表达，还需要最低数量的血小板维持血管屏障。血小板疾病分为血小板数量减少和功能异常，病因可以是遗传性、获得性。与血小板相关的出血可以是轻微至危及生命的出血。

IPFD的病因包括黏附蛋白受体缺陷、可溶性激动剂受体缺陷、血小板颗粒异常、信号传导通路异常、膜磷脂缺陷等[1]，其中，信号传导通路异常被认为是最常见的病因。临床表现为不同严重程度的出血倾向、诊断困难。近年来，随着基因测序技术的发展，越来越多的致病基因被发现，基因筛查提高了这类疾病的诊断率。2014年Canault等[2]首次报道RASGRP2基因突变导致血小板中Rap1激活并发生严重出血的病例，发现了RASGRP2基因中的致病突变（c.G742T），该基因编码钙和甘油二酯调节鸟嘌呤交换因子Ⅰ（CalDAG-GEFI），携带该突变的个体血小板激活Rap1的能力下降，且αⅡbβ3整合素的“由内向外”信号传导功能异常。此后，国内外均有RASGRP2基因突变导致IPFD的病例报道[3]–[6]。

本例患儿自幼鼻腔异常出血，血小板计数、形态及凝血功能正常，血小板膜糖蛋白轻度异常，血小板聚集异常，RASGRP2基因c.74-1G>C（p.?）致病性纯合变异，父母该位点杂合变异，诊断为遗传性血小板功能障碍。

RASGRP2致病性变异致CalDAG-GEFI蛋白表达缺失或功能障碍，RAP1和整合素激活缺陷，并持续性降低血小板对弱激动剂（如二磷酸腺苷、肾上腺素及低浓度胶原等）的聚集反应，引起一种常染色体隐性遗传非综合征性血小板功能异常，被称为遗传性血小板功能异常18型（IPFD-18）。RASGRP2基因编码的CalDAG-GEFI蛋白仅负责介导血小板受弱激动剂刺激后的活化信号传导，不影响整合素αⅡbβ3本身的结构和结合功能。血栓弹力图的最大振幅（MA）主要评估血小板通过整合素αⅡbβ3与纤维蛋白原交联形成血凝块的强度。本病例整合素αⅡbβ3功能正常，能正常结合纤维蛋白原，故血栓弹力图MA正常，血小板聚集试验在二磷酸腺苷和胶原蛋白诱导下受损。

IPFD的治疗措施包括避免使用阿司匹林等干扰血小板功能的药物，鼻腔填塞等局部止血治疗，抗纤维蛋白溶解剂、去氨加压素、重组活化因子Ⅶa、血小板输注等全身促止血治疗。allo-HSCT与基因治疗可治愈本病，但需注意并发症，权衡利弊。查阅文献未发现allo-HSCT治疗RASGRP2突变相关IPFD的报道。allo-HSCT已成功用于治疗血小板无力症[7]–[11]，除1例采用HLA5/10相合亲缘供者外，其余均为HLA全相合供者，预处理方案均为清髓性，均顺利植入，原发病治愈，2例出现GVHD、病毒感染、自身免疫性溶血性贫血等并发症（表1）。

**表1:** 近5年接受异基因造血干细胞移植治疗的血小板无力症病例

作者	例数	供者来源及HLA配型	预处理方案	粒细胞植入时间（d）	血小板植入时间（d）	嵌合情况	GVHD/其他并发症	随访时间（月）及结果
Palma-Barqueros等[1]	1	MUD，9/10	BU/CY	10	11	完全嵌合	无	6，存活
韩怡天等[7]	2	MRD，10/10；MMRD，5/10	BU/CY；FLU/CY	10；10	63；10	完全嵌合；完全嵌合	无	42，存活；40，存活
Canault等[2]	1	MRD，10/10	BU/CY	11	11	完全嵌合	无	20，存活
Shi等[3]	2	MRD，10/10；MUCB，6/6	BU/CY；BU/CY	18；40	22；33	完全嵌合；完全嵌合	有/CMV感染；无/AIHA	60，存活；67，存活

**注** MUD：匹配的无关供者；MRD：匹配的亲缘供者；MMRD：不匹配的亲缘供者；MUCB：匹配的无关脐带血供者；BU：白消安；CY：环磷酰胺；FLU：氟达拉滨；GVHD：移植物抗宿主病；CMV：巨细胞病毒；AIHA：自身免疫性溶血性贫血

本例患儿为新发RASGRP2突变相关IPFD，反复出现危及生命的出血，同胞姐姐因大量出血死亡，患儿父母为根治疾病要求行allo-HSCT。因有HLA全相合且未携带致病突变的同胞供者，allo-HSCT技术成熟，权衡利弊后采用allo-HSCT治疗。考虑到该类疾病骨髓造血及免疫功能正常，本例患儿及文献资料均采用清髓性预处理方案及常规GVHD预防方案，本例患儿顺利植入，未出现移植相关并发症，造血重建后未再发生出血，检测嵌合率为完全供者型，血小板聚集试验胶原诱导下仍有聚集率降低，考虑可能与骨髓造血功能未完全恢复有关，需动态监测。

随着对遗传病基因诊断的重视程度逐渐加深及基因测序技术的不断进步，新发致病突变逐渐被发现，为IPFD的基因治疗建立了基础。基因治疗用于IPFD目前仍处于研究和临床试验阶段，其长期疗效和安全性需进一步观察和评估。

allo-HSCT是治愈IPFD的有效手段，应注意有创操作时及血小板减少期间的出血风险，本例患儿通过输注血小板、鼻腔填塞等手段度过高风险出血阶段。另需注意评估风险与收益，尤其在无良好供者时，应评估allo-HSCT相关并发症带来的风险，选择患儿获益最大的治疗手段。
